# Functional Clustering of Periodic Transcriptional Profiles through ARMA(*p*,*q*)

**DOI:** 10.1371/journal.pone.0009894

**Published:** 2010-04-16

**Authors:** Ning Li, Timothy McMurry, Arthur Berg, Zhong Wang, Scott A. Berceli, Rongling Wu

**Affiliations:** 1 Department of Epidemiology and Biostatistics, University of Florida, Gainesville, Florida, United States of America; 2 Department of Mathematical Sciences, DePaul University, Chicago, Illinois, United States of America; 3 Division of Biostatistics, Pennsylvania State University College of Medicine, Hershey, Pennsylvania, United States of America; 4 Center for Computational Biology, Beijing Forestry University, Beijing, People's Republic of China; 5 Department of Surgery, University of Florida, Gainesville, Florida, United States of America; University of Manchester, United Kingdom

## Abstract

**Background:**

Gene clustering of periodic transcriptional profiles provides an opportunity to shed light on a variety of biological processes, but this technique relies critically upon the robust modeling of longitudinal covariance structure over time.

**Methodology:**

We propose a statistical method for functional clustering of periodic gene expression by modeling the covariance matrix of serial measurements through a general autoregressive moving-average process of order (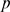,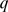), the so-called ARMA(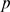,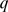). We derive a sophisticated EM algorithm to estimate the proportions of each gene cluster, the Fourier series parameters that define gene-specific differences in periodic expression trajectories, and the ARMA parameters that model the covariance structure within a mixture model framework. The orders 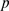 and 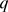 of the ARMA process that provide the best fit are identified by model selection criteria.

**Conclusions:**

Through simulated data we show that whenever it is necessary, employment of sophisticated covariance structures such as ARMA is crucial in order to obtain unbiased estimates of the mean structure parameters and increased precision of estimation. The methods were implemented on recently published time-course gene expression data in yeast and the procedure was shown to effectively identify interesting periodic clusters in the dataset. The new approach will provide a powerful tool for understanding biological functions on a genomic scale.

## Introduction

DNA microarray technologies are widely used to detect and understand genome-wide gene expression regulation and function. Microarray experiments typically collect expression data on thousands of genes and the high dimensionality of the data impose statistical challenges. The statistical issues become even more pronounced when transitioning from static microarray data to *temporal* microarray experiments where the gene expression levels are traced over a period of time. Examples of temporal microarray experiments include studies of the cell cycles in yeast [1] and the circadian cycles in mice [2]. It is well known that a lot of biological processes are characterized by periodic rhythms as a result of nonlinear cellular regulation, such as the aforementioned circadian rhythms in mice, cell division [3], and complex cell cycles in some organisms [4], [5]. The temporal microarray experiments are useful in understanding the periodicity and regulation of behavioral and physiological rhythms in organisms, and through clustering gene expression profiles based on their periodic patterns, it is possible to associate genes with physiological functions of interest. Functional principal component analysis and mixture models have become popular dimension reduction tools in microarray studies to cluster genes of similar temporal patterns [6]–[12]. These methods model the time-dependent gene expression profiles based on nonparametric approaches. The proposed model models the expression profiles by a Fourier series which can be considerably more powerful in the presence of truly periodic signals while remaining robust to non-periodic signals. This is illustrated in the real data analysis in the Real Data Application section below.

There has been a long history of using parsimonious mathematical functions, e.g. the Fourier series, to describe periodic biological processes [13], [14]. Recent application of the Fourier series approximation lies in the areas of identification of patterns of biological rhythmicity during the neonatal period [15], pharmacodynamics [16] and detection of periodic gene expression in various organisms [1], [17]–[19]. Kim et al. [20] integrated the Fourier series approximation into a mixture model approach to functional clustering of gene expression on the basis of their periodic patterns, which makes it possible to test biologically meaningful characteristics of expression profiles such as the differences in gene expression trajectories, curve features, and the duration of biological rhythms.

Although the approach proposed by Kim et al. [20] efficiently enhances the model power by assuming the first-order autoregressive (AR(1)) covariance structure for the time-dependent gene expression data, such approximation may not always be adequate in real practice. The autoregressive moving-average model, which is usually referred to as ARMA(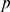,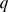), has been commonly used in time series analysis and is viewed as a higher order and thus more flexible class of covariance structures than AR(1) [21]–[23]. It is generated from an autoregressive (AR) process of order 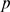 and a moving average (MA) process of order 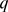; the AR(1) model is a special case of the ARMA(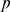,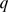) model with 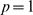 and 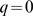. In this article, we extend the approach of Kim et al. [20] by using the more flexible ARMA(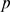,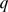) covariance structure for the gene expression profiles.

Unlike AR(1), the ARMA covariance matrix generally does not have closed form solutions for its inverse and determinant, which imposes challenges in parameter estimation and likelihood function evaluation. We use a recursive method [24] and a numerical differentiation approach [25] to evaluate the likelihood function and estimate the covariance parameters in the ARMA(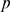,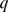) model. However, the computational burden and complexity increase dramatically compared to the closed form model of Kim et al. (2008), though on a modern computer these calculations still remain very reasonable.

The rest of the article is organized as follows. The model and the inference procedure are described in Section 2. Section 3 includes simulation studies to investigate the improvement in estimation accuracy and efficiency comparing the ARMA (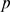,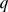) with AR(1). Discussions and further remarks are provided in the last section.

## Methods

### Mixture Model

We consider a finite mixture model for clustering the gene expression profiles of 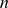 genes. For a detailed discussion of the finite mixture models, a suggested reference is [26]. We assume the genes are measured at equally spaced time points 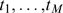, where 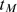 is the longest possible observation time. The individual genes may have fewer than 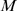 measurements, and for simplicity, we assume there is no missing data in between two observed measurements. Let the vector 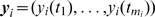 collect the expression data for gene 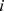 over the 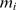 time points, where 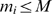. We assume there are 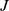 expression patterns in the 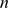 genes, which indicates that there are 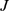 components in the mixture model and each gene arises from one and only one of the 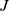 possible components. We further assume that 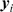 is a realization of a mixture of 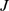 multivariate normal distributions with the density function specified as
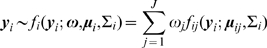
(1)where 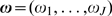 is a vector of non-negative proportions for the 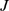 patterns that sum to unity and 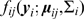 denotes the density function for the 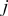-th gene expression pattern, a multivariate normal with mean vector 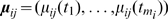 and the common 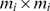 covariance matrix 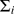. Let 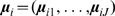 contain the pattern-specific mean vectors for gene 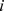.

The Fourier series can be used to approximate time-dependent expression if the genes are periodically regulated (Spellman et al. 1998). It decomposes the periodic expression level into a sum of the orthogonal sinusoidal terms. The general form of the Fourier signal is
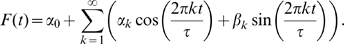
(2)The coefficients 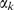 and 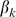 determine the times at which the expression level achieves maximums and minimums, 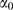 is the average expression level of the gene, and 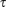 specifies the periodicity of the regulation. The gene expression value over time can be approximated by partial sum of the Fourier series decomposition where the sum in (2) only contains 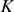 terms. We denote this Fourier series approximation by 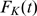; specifically,
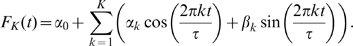
For pattern 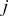, the mean expression value of gene 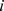 at time 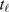, 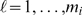, is 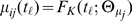, where 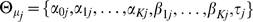 denotes the vector of Fourier parameters of the first 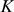 orders. To put the mean structure into the normality framework specified in (1), we assume that for gene 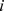, if it belongs to pattern 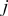, the observed data are 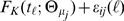 for 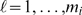, where the random errors are components of a multivariate normal distribution; i.e.,
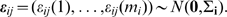
A common and convenient method to model the covariance structure of 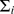 is to use the first-order autoregressive model (AR(1)). Although the AR(1) covariance matrix has computational advantages through having closed form expressions of its inverse and determinant, it lacks flexibility being parameterized by only two parameters (typically denoted by 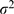 and 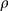). In order to accommodate more robust covariance structures, we adopt a flexible approach using the autoregressive moving-average process, ARMA(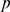,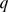) [23]. The zero-mean random error 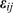 is generated according to the following process
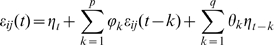
where 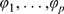 and 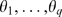 are unknown parameters, and 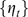 is a sequence of independent and identically distributed (iid) normal random variables with zero mean and variance 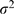. Certain restrictions are imposed on the parameters of the ARMA model to insure estimability; further details can be found in [24] and [27]. The ARMA(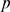,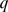) model parameters are listed in 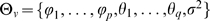.

The total number of parameters to be estimated with 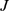 clusters, an ARMA(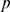,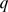) covariance structure, and a Fourier series of degree 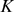 comes to 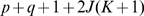.

### Likelihood and Algorithm

Denote the entire set of unknown parameters as 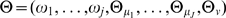 denote the set of unknown parameters in the mixture model. In the absence of knowledge on the membership of the expression pattern for the genes, the likelihood function based on the mixture model (1) is
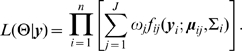
The log-likelihood function is non-linear in 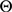 which imposes difficulty in estimating the unknown parameters. Here we use the EM algorithm to obtain the maximum likelihood estimate of 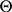. Let 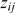 be a latent variable, defined as 1 if gene 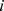 arises from the 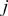-th pattern, and write 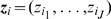. Then 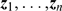 are 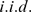 multinomial random variables with probabilities 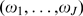. The complete data log-likelihood is thus
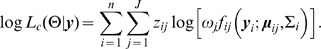
(3)In the E-step of the algorithm, the posterior expectation of 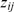, i.e., the posterior probability that gene 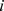 arises from the 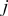-th pattern, is evaluated given the current estimate of 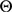 and the data. In the M-step, 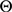 is updated from the expectation of complete data log-likelihood in which 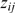 is replaced by its posterior expectation from the E-step. The algorithm proceeds by iterating between the two steps until convergence. The details of the EM algorithm are given in the Supporting Information (Text S1).

### Model Selection

Our mixture model assumes that the number of components in the mixture model (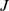) and the order of 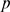 and 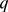 in the ARMA covariance structure in 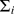 are known before estimation of the parameters. In practice, however, the model that provides the best fit to the data in terms of 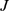, 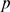, and 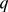 can be identified using the Akaike information criterion (AIC) [28] and the Bayesian information criterion (BIC) [29], which are defined as follows:
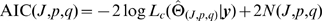


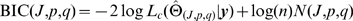
where 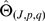 is the maximum likelihood estimate of 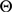 and it is indexed by 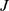 and 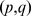, and 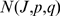 is the number of parameters in the mixture model determined by 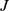 and 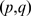. The selected model has the smallest AIC and BIC.

Under the framework of maximum likelihood estimation, it is possible that the likelihood increases when more parameters are added into the model, which could lead to overfitting. Both AIC and BIC resolve this problem by including a penalty term for the number of parameters, but BIC imposes a stronger penalty than AIC, and as a result, it tends to select models with smaller number of parameters than those chosen by AIC method.

The dimension of our model parameters can be viewed as growing in two directions, one determined by 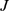 and the other by 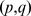. A one unit increase in 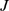 gives arise to addition of 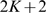 parameters, which is always larger than a one unit increase in 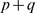, we propose a three-step procedure to select the best model. First, we fit an ARMA covariance structure with relatively low orders 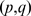 to 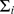, i.e., ARMA(1,0) or ARMA(1,1), and calculate AIC or BIC values by varying 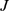 starting from 1. The model with the smallest AIC or BIC is identified. We denote the corresponding 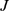 as 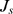. We then fit the mixture model with 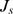 components, but this time vary 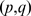 to find the best combination 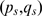. In the third step, we go back to step 1 and refit the model with ARMA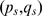 and select 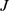 again. The resulting model with the smallest AIC or BIC is our final choice. Alternative to the three-step procedure, if the amount of computation is not a limiting factor, one could simply calculate the AIC or BIC values for all models under consideration and select the model that minimizes the criterion of choice.

### Hypothesis Tests

The existence of at least two different transcriptional expression profile patterns over the 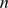 genes under study can be tested by formulating the following hypothesis:
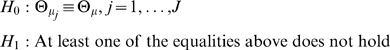
(4)where 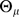 is the vector of the Fourier series parameters when gene expression pattern-specific differences do not exist for the given data. The likelihood ratio test statistic can be calculated under the null and alternative hypotheses; that is,
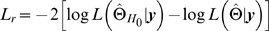
where 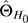 and 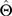 stand for the MLEs of the parameters under the null hypothesis and the alternative, respectively.

Since there is no closed-from distribution for 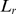, the critical value for claiming the existence of at least two different expression patterns is determined by a parametric bootstrap method. We simulate 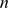 gene expression profiles at the observed time points under the multivariate normal model indicated by the null hypothesis. The true values of the parameters in the simulation are taken to be the MLE's under the null hypothesis, i.e., 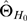. For each simulated dataset, the likelihood ratio test statistic 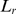 is calculated by fitting the models under the null and the alternative hypotheses. This procedure is repeated for a large number of times, say 1000, and the 95th percentile of the empirical distribution of 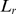 is then regarded as the critical value of the test (4).

## Results

### Simulation Results

The performance of the proposed mixture model in terms of the precision and efficiency of the parameter estimates and the model selection for the number of components have been extensively studied in [20], where the AR(1) covariance structure was considered for 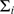. Kim et al. show that the mixture model and the EM algorithm can provide reasonably precise estimates of all parameters and AIC and BIC are able to select the right number of components 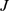 in the model. The model was also compared with the random-effect mixture model proposed by [11] and biased parameter estimates were observed for Ng et al.'s method when the gene-expression profiles follow Fourier series approximations.

**Figure 1:**
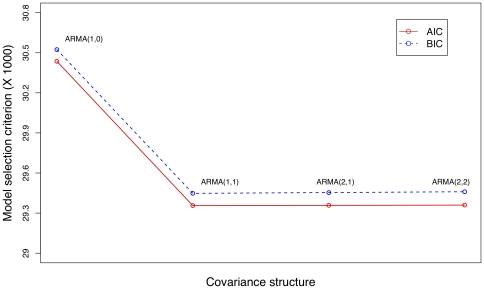
In the first simulation study, AIC and BIC values calculated using a simulated dataset whose true covariance structure is ARMA (1,1) to identify the optimal covariance structure to be used.

In this article, we focus on the influence of the assumed covariance structure on the estimation of the proportion parameter 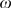 and the mean structure parameters 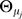, 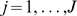. We generated 400 genes from three distinct expression patterns, and the expression of each gene was measured at 25 equally spaced time points. The mean of expression values was simulated from a second-order Fourier series. In the first set of simulations, the true covariance structure for the time-dependent expression was ARMA(2,2), but the data were analyzed using ARMA(2,2), ARMA(2,1), ARMA(1,1) and ARMA(1,0), as shown in Tables 1, 2, 3, and 4, respectively. When the assumed covariance structure is the correct one (Table 1), the approach produces relatively accurate estimates for all parameters, but less sufficiently sophisticated covariance structures could lead to large bias and loss of efficiency in estimation of 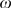 and 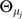, 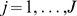 (Tables 2, 3 and 4). We further simulated gene expression profiles under the covariance structure ARMA(1,0), and obtained sound parameter estimates when the data were analyzed using ARMA(2,2) (Table 5). And finally, using a simulated dataset with the true covariance ARMA(1,1), we show that the order p and q in the ARMA covariance structure can be correctly determined by AIC and BIC values (Figure 1).

**Table 1:** Simulated averages and standard errors of parameter estimates using the ARMA(2,2) model when the true covariance structure is ARMA(2,2) (No. simulations = 200).

	Pattern		
	1	2	3
Proportion			
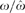	0.300/0.301(0.022)	0.500/0.497(0.027)	0.200/0.202(0.022)
Mean vector			
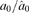	2.000/1.999(0.234)	2.050/2.032(0.183)	2.100/2.121(0.312)
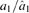	0.500/0.495(0.074)	−0.400/−0.393(0.060)	0.600/0.606(0.115)
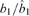	−0.800/−0.803(0.062)	0.700/0.697(0.063)	−0.700/−0.713(0.105)
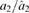	−0.500/−0.497(0.035)	−0.600/−0.601(0.031)	−0.500/−0.501(0.054)
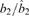	1.000/1.004(0.025)	1.100/1.099(0.026)	1.000/1.000(0.040)
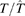	120.000/120.020(0.190)	135.000/134.992(0.197)	140.000/140.037(0.426)
Covariance			
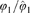		1.300/1.300(0.016)	
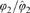		−0.400/−0.401(0.015)	
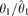		0.700/0.700(0.017)	
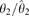		0.120/0.118(0.011)	
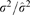		0.700/0.698(0.010)	

**Table 2:** Simulated averages and standard errors of parameter estimates using the ARMA(2,1) model when the true covariance structure is ARMA(2,2) (No. simulations = 200).

	Pattern		
	1	2	3
Proportion			
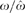	0.300/0.301(0.022)	0.500/0.496(0.028)	0.200/0.202(0.022)
Mean vector			
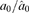	2.000/1.995(0.234)	2.050/2.031(0.175)	2.100/2.112(0.317)
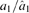	0.500/0.496(0.072)	−0.400/−0.394(0.060)	0.600/0.600(0.109)
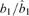	−0.800/−0.804(0.060)	0.700/0.696(0.064)	−0.700/−0.711(0.107)
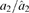	−0.500/−0.497(0.035)	−0.600/−0.600(0.031)	−0.500/−0.501(0.053)
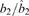	1.000/1.004(0.025)	1.100/1.099(0.026)	1.000/1.002(0.040)
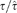	120.000/120.020(0.195)	135.000/134.987(0.194)	140.000/140.037(0.415)
Covariance			
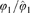		1.300/1.397(0.007)	
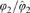		−0.400/−0.488(0.005)	
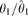		0.700/0.585(0.009)	
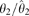		0.120/-	
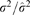		0.700/0.701(0.010)	

**Table 3:** Simulated averages and standard errors of parameter estimates using the ARMA(1,1) model when the true covariance structure is ARMA(2,2) (No. simulations = 200).

	Pattern		
	1	2	3
Proportion			
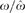	0.300/0.301(0.022)	0.500/0.491(0.029)	0.200/0.207(0.025)
Mean vector			
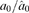	2.000/1.997(0.244)	2.050/2.059(0.185)	2.100/2.023(0.311)
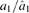	0.500/0.494(0.073)	−0.400/−0.430(0.067)	0.600/0.664(0.122)
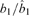	−0.800/−0.803(0.062)	0.700/0.734(0.069)	−0.700/−0.769(0.104)
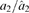	−0.500/−0.498(0.036)	−0.600/−0.595(0.032)	−0.500/−0.491(0.053)
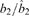	1.000/1.004(0.025)	1.100/1.098(0.027)	1.000/0.997(0.040)
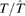	120.000/120.018(0.196)	135.000/135.067(0.196)	140.000/139.808(0.482)
Covariance			
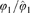		1.300/0.927(0.002)	
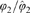		−0.400/-	
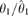		0.700/0.803(0.006)	
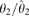		0.120/-	
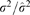		0.700/0.839(0.014)	

**Table 4:** Simulated averages and standard errors of parameter estimates using the ARMA(1,0) model when the true covariance structure is ARMA(2,2) (No. simulations = 200).

	Pattern		
	1	2	3
Proportion			
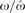	0.300/0.301(0.022)	0.500/0.443(0.092)	0.200/0.256(0.091)
Mean vector			
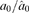	2.000/2.014(0.247)	2.050/2.112(0.191)	2.100/1.925(0.283)
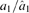	0.500/0.484(0.097)	−0.400/−0.333(0.414)	0.600/0.547(0.406)
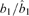	−0.800/−0.796(0.112)	0.700/0.591(0.521)	−0.700/−0.564(0.514)
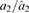	−0.500/−0.494(0.073)	−0.600/−0.572(0.056)	−0.500/−0.494(0.069)
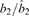	1.000/0.991(0.088)	1.100/1.088(0.052)	1.000/1.006(0.055)
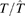	120.000/119.838(5.409)	135.000/135.718(1.487)	140.000/138.907(1.552)
Covariance			
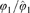		1.300/0.952(0.002)	
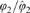		−0.400/-	
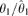		0.700/-	
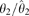		0.120/-	
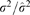		0.700/1.732(0.057)	

**Table 5:** Simulated averages and standard errors of parameter estimates using the ARMA(2,2) model when the true covariance structure is ARMA(1,0) (No. simulations = 200).

	Pattern		
	1	2	3
Proportion			
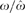	0.300/0.294(0.060)	0.500/0.490(0.098)	0.200/0.216(0.111)
Mean vector			
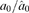	0.500/0.515(0.123)	0.400/0.390(0.088)	0.600/0.600(0.304)
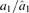	−0.500/0.503(0.082)	0.300/0.311(0.056)	0.200/0.189(0.168)
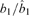	0.400/0.395(0.094)	−0.200/−0.202(0.064)	0.200/0.201(0.230)
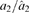	0.100/0.095(0.035)	0.150/0.148(0.032)	0.050/0.063(0.117)
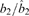	0.050/0.052(0.040)	0.070/0.071(0.045)	0.100/0.086(0.113)
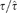	120.000/119.953(1.619)	135.000/134.901(1.658)	150.000/151.137(12.234)
Covariance			
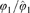		0.800/0.797(0.008)	
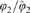		0/−0.001 (0.001)	
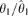		0/0.003 (0.013)	
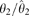		0/−0.001 (0.002)	
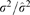		1.000/0.996(0.016)	

Further simulations were performed to investigate the effects of 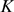 on the estimation. Indeed there is a balance between choosing a sufficiently large 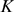 so as to accurately the model periodic mean curve without selecting too large of a 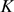 where the model would be overfit. Indeed, as described above, the AIC and BIC can assist in selecting the order 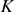, but together with selecting 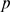, 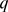, and 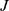, computations can be somewhat burdensome. In practice, 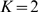 or 3 should nicely model fairly intricate periodic expressions. To further test the effectiveness of the methods, we performed a second set of simulations and compared the results of using 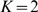 with 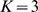 and measured their performance with the adjusted Rand index.

**Figure 2:**
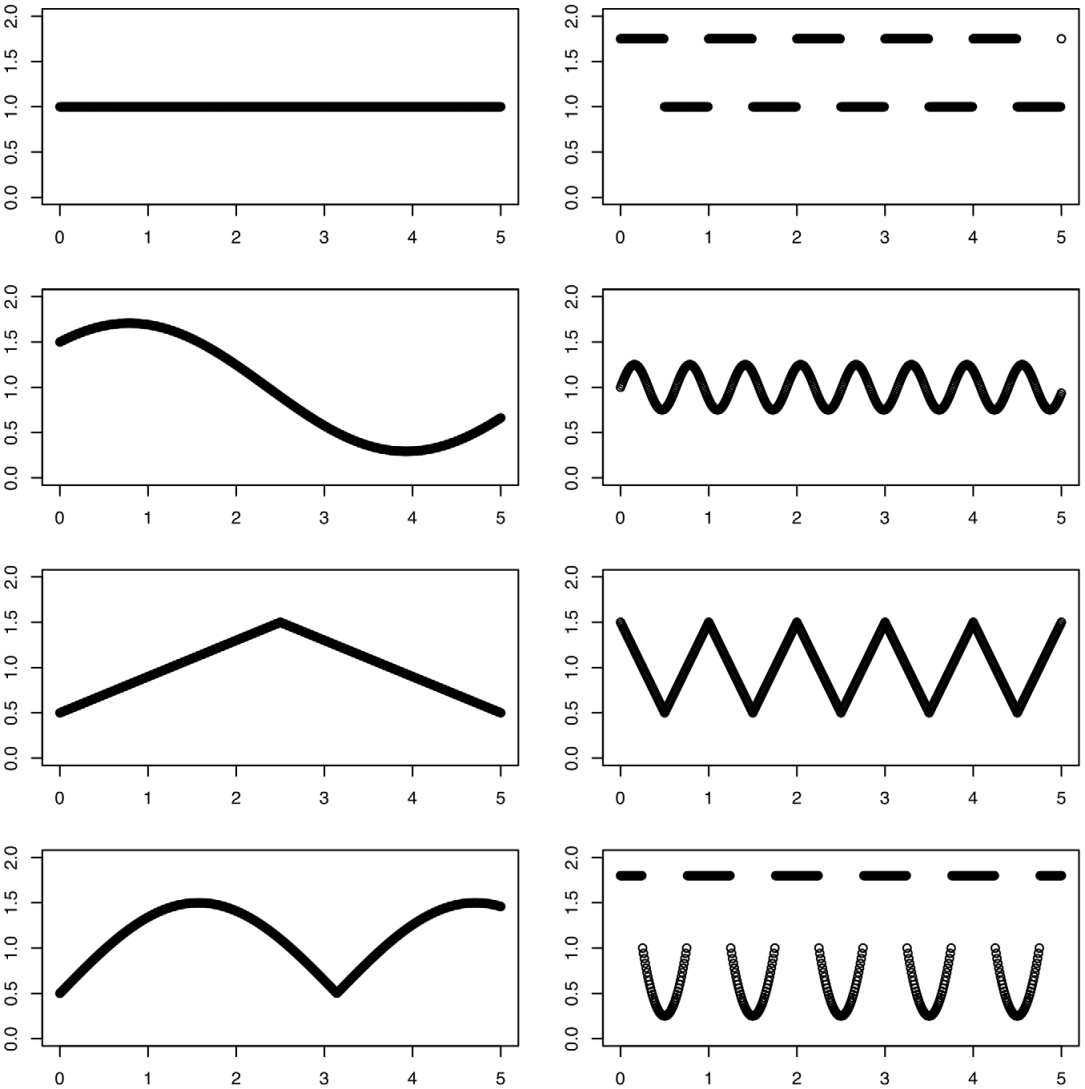
Simulations were performed using time-course expression data with 40 time points and 100 genes per cluster simulated from eight mean curves graphed here.

**Figure 3:**
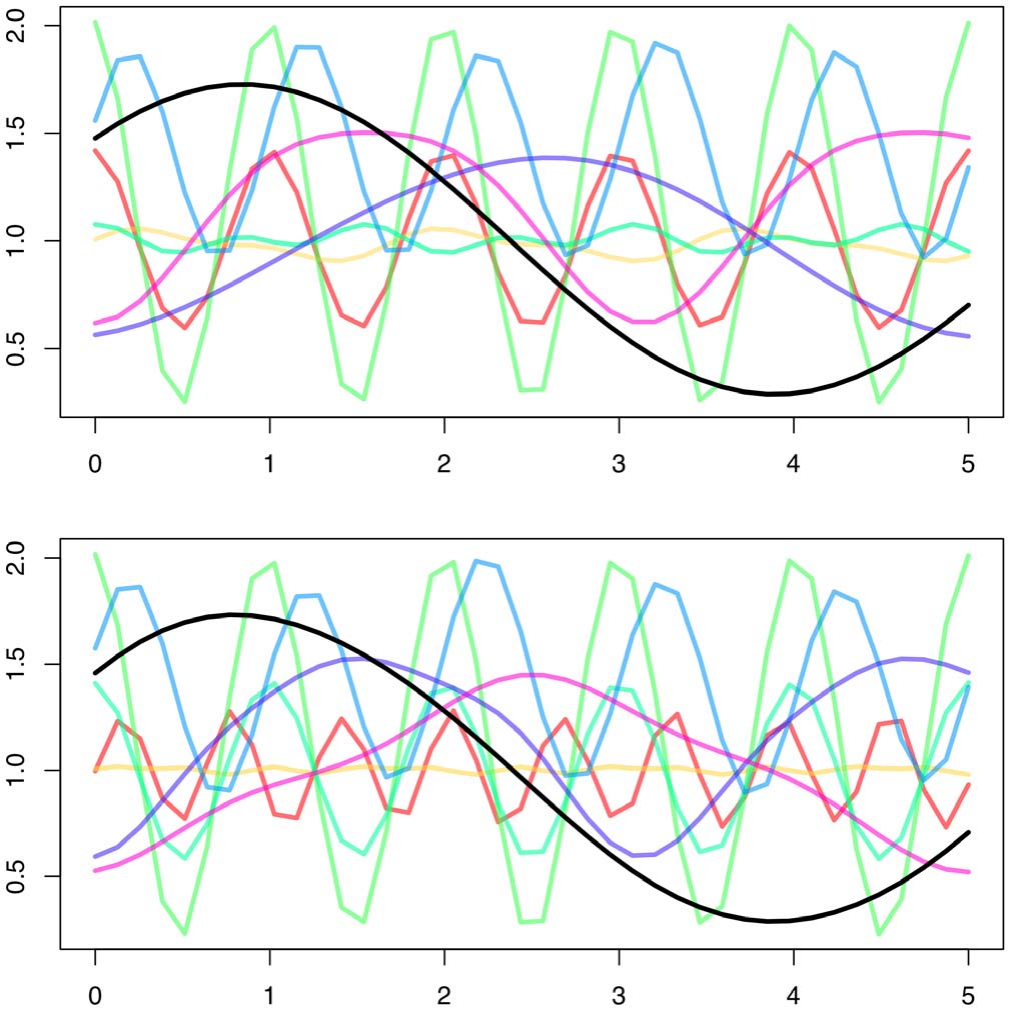
Estimated mean curves estimated from the simulated data with parameters 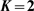 (top) and 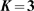 (bottom).

Eight time-course expression profiles were simulated with the mean expression profiles graphed in Figure 2. In total, 800 genes consisting of 100 genes per cluster were simulated with 40 equally spaced time points. Stationary noise generated from an AR(2) model and standard deviation approximately equal to .3 was added to the simulated data. Function clustering with AR(2) covariance structure was performed, and the resulting estimated mean curves were graphed in Figure 3 with 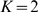 (top graph) and 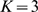 (bottom graph). From the mean curves, we see that seven of the eight clusters were correctly identified, and with 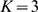, all eight mean curves were correctly identified. The simulation performance is further quantified by the adjusted Rand Index as implemented in *mclust* R package [30]. For estimation with 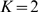, the adjusted Rand index is .825 (the larger the better), and it is .964 for estimation with 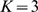.

**Figure 4:**
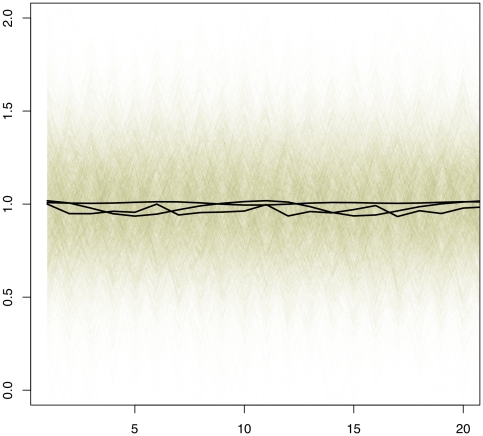
Functional clustering was applied to stationary noise following an AR(2) covariance structure. AIC and BIC selected three mean curves and the illustrated mean profiles are small compared with the variation of the data as drawn in the background.

Another set of simulations were performed to identify the types of clusters that would be estimated on data generated without any signals, that is, data generated from pure noise. Stationary noise following an AR(2) process was simulated and the model was used to identify periodic clusters. Here, the AIC and BIC selected three clusters, but the mean functions for the three clusters are all nearly zero compared to the standard deviation of the noise (approximately .3). This is illustrated in Figure 4 where all gene expression profiles are drawn in the background and mean curves, assuming three clusters, are graphed in black. The small amplitudes of the mean curves suggest the three clusters are in fact simply clustering the noise. The weak clustering is further illustrated in Table 6, by varying thresholds between 10% and 99% to investigate its effect on the resulting cluster sizes. Two clusters had very weak clustering and the third cluster essentially clustered the entire dataset.

**Table 6:** Effects of classification threshold on cluster sizes for the three cluster estimation on simulated noise.

threshold	#1	#2	#3
**.95**	0	0	0
**.90**	0	0	125
**.80**	0	0	1804
**.70**	0	0	2774
**.60**	0	0	2952
**.50**	0	0	2952
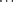	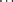	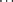	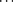
**.20**	130	21	2955
**.10**	1168	1065	2955

### Real Data Application

This methodology is applied to time time course gene expression data published in [31]. For their research, a total of 8 time-course experiments were performed with expression data collected at 18 to 22 times on 15-minute intervals. We analyzed data from one time-course experiment where the original and processed data is accessible from ArrayExpress with accession number E-MEXP-54. Approximately 3000 genes over 21 time measurements were considered for application of our methods.

**Figure 5:**
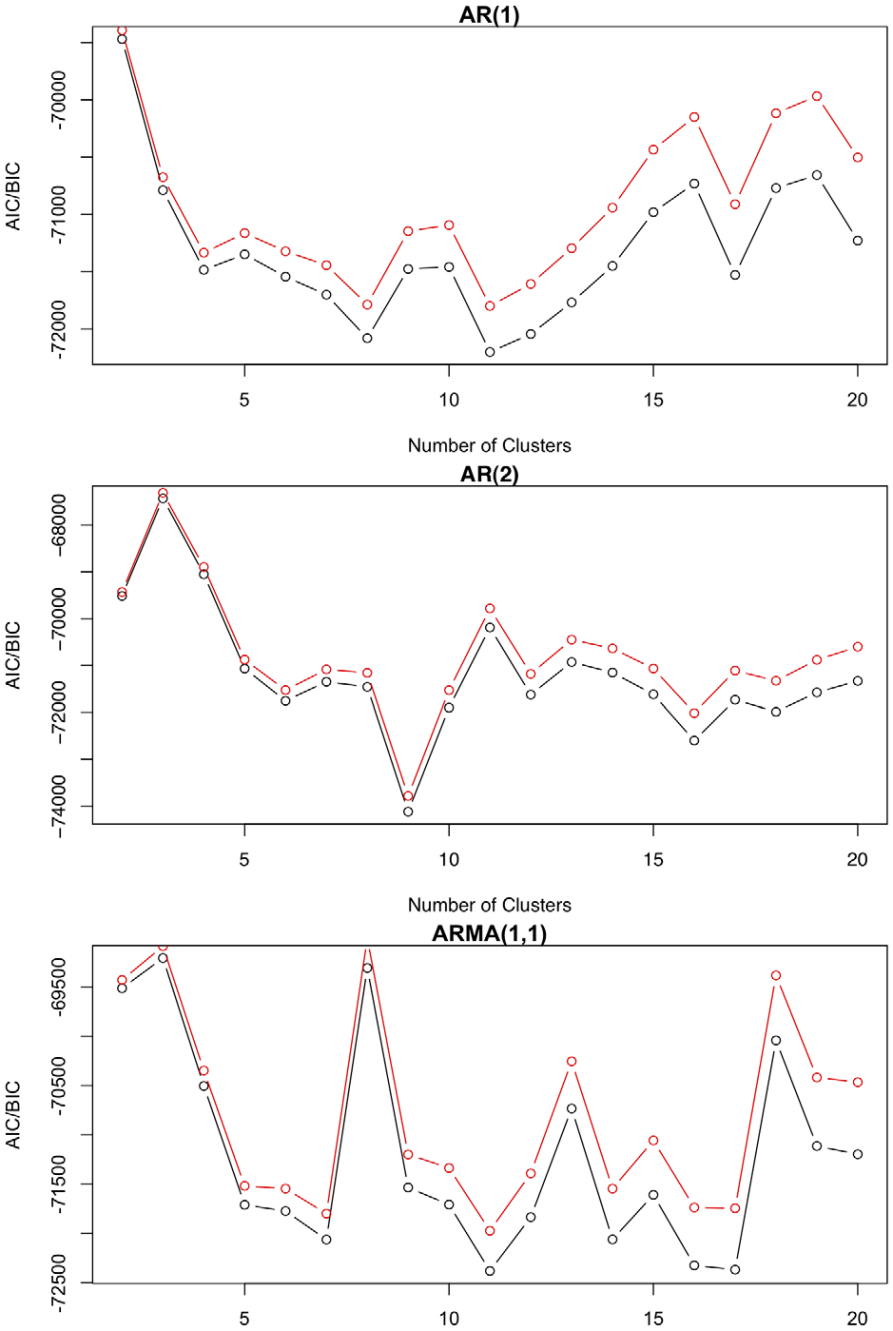
In real data application, AIC and BIC values for different ARMA structures are calculated over varying number of cluster sizes (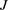). The ARMA model with smallest AIC/BIC turned out to be the AR(2) model.

To keep the model relatively parsimonious, a single covariance structure was used to model the collection of genes, i.e. 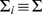, and with the following models: ARMA(1,0), ARMA(2,0), and ARMA(1,1). Additionally, allowing for a robust periodic fit but with keeping number of parameters reduced, Fourier series of order two was fit to all of the clusters. The initial values of the parameters in the EM algorithm were randomly selected from reasonable ranges as suggested by the expression profiles. A so-called absorption cluster was also included to soak up the less informative genes with no signal in their time-course profiles; this cluster was initiated in the EM algorithm with zero amplitude. The AIC and BIC values for varying number of clusters across the three covariance structures are graphed in Figure 5. The minimum AIC and BIC values under each covariance structure with corresponding number of clusters are reported in Table 7. The overall smallest AIC and BIC was obtained with the ARMA(2,0), or just simply AR(2), covariance structure identifying 9 distinct clusters that includes the absorption cluster. The estimated ARMA parameters are 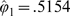, 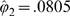, and 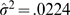.

**Table 7:** Minimum AIC and BIC values, as well as the corresponding optimal number of clusters, over varying number of clusters for the ARMA(1,0), ARMA(1,1), and ARMA(2,0) covariance structures.

Covariance Structure	AIC	BIC	# of clusters
ARMA(1,0)	−72200.16	−71798.74	11
ARMA(1,1)	−72379.89	−71972.49	11
ARMA(2,0)	**−74119.34**	**−73783.83**	9

**Figure 6:**
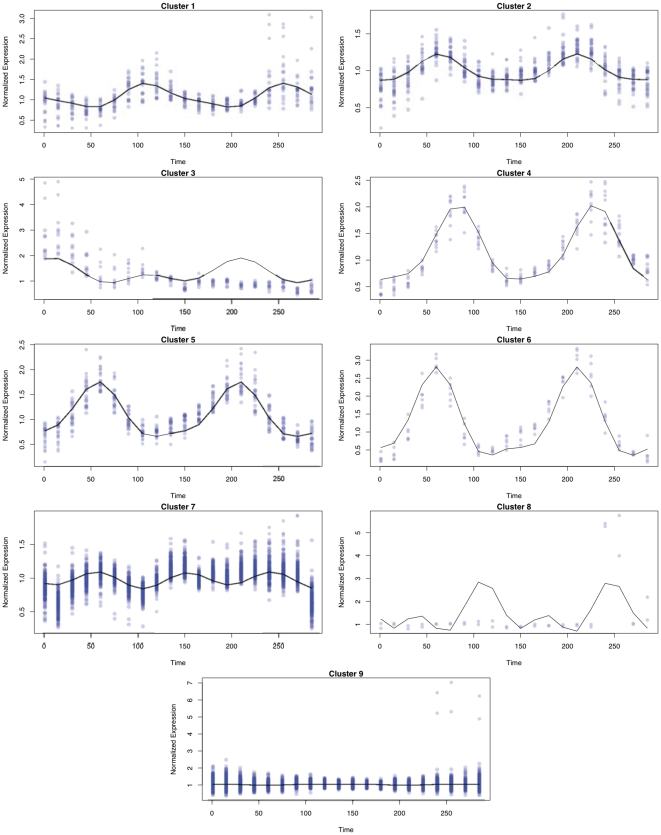
Mean curves for each of the 9 clusters identified are individually graphed together with time-course gene expression profiles of genes classified to the cluster. A gene is classified to the cluster if it has greater than a 90% probability of belonging to the cluster.

Genes are classified to the cluster if they have an estimated probability of 90% or greater of belonging to the cluster. The mean functions for the identified nine distinct clusters are depicted in Figure 6 together with expression profiles of the genes that are classified to the cluster. In this figure, we see the clustering approach is very effective at identifying tightly coupled clusters, even when genes within the cluster don't elegantly follow a periodic structure as seen in clusters 3 and 8. The threshold of 90% is somewhat arbitrarily chosen, and we consider varying thresholds between 10% and 99% to investigate its effect on the resulting cluster sizes. The results are tabulated in Table 8. Clusters 3, 4, 5, 6, 8 remain fairly stable in that they only consist of strongly classified genes, whereas the other clusters have a mix of strongly classified genes and weakly classified genes. Under the 90% threshold, the absorption cluster (the cluster with small variation in expression) soaked up approximately 72% of the genes (2142 genes), and 17% of the genes (501 genes) did not have a dominating cluster defined by the 90% or greater estimated probability threshold. Many genes and their periodic expressions were shown to be effectively clustered by this model.

**Table 8:** The effects of classification threshold on cluster sizes.

threshold	#1	#2	#3	#4	#5	#6	#7	#8	#9
**.99**	11	12	9	10	18	6	86	2	1678
**.95**	14	20	9	10	19	6	173	2	2010
**.90**	19	27	10	10	21	6	217	2	2142
**.80**	25	36	10	10	21	6	275	2	2258
**.70**	28	41	10	10	22	6	303	2	2333
**.60**	30	46	10	10	22	6	357	2	2384
**.50**	31	47	10	10	22	6	395	2	2416
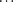	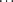	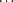	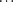	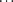	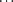	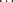	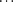	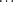	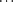
**.10**	40	97	10	10	22	6	668	2	2600

**Figure 7:**
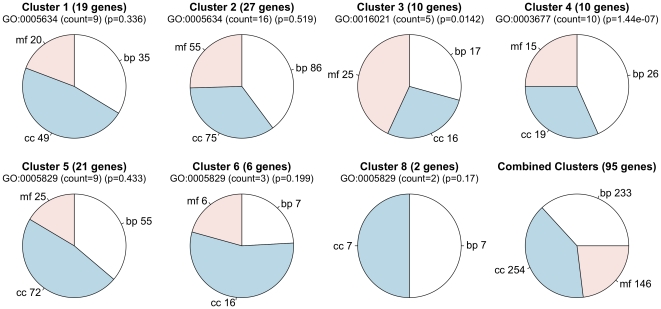
GO analysis of seven clusters identified in the real data analysis. Pie charts depict the distribution of biological process (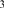
bp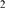), cellular component (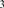
cc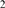), and molecular function (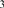
mf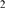) in each of the clusters. The most prevalent GO category is indicated below the title with number of times it is present and a p-value computed from a hypergeometric test. The GO listed categories include (GO:0005634, “nucleus”; GO:0016021, “integral to membrane”; GO:0003677, “DNA binding”; GO:0005829, “cytosol”).

The analysis of the real data set did suggest some interesting clusters, and we performed a gene ontology (GO) analysis on the tight clusters (clusters 3, 4, 5, 6, and 8) along with clusters 1 and 2. Basic GO organization consisting of three major categories – “biological process”, “cellular component”, and “molecular function” – is considered in addition to a more specific GO classification. Figure 7 depicts the seven clusters, together with all clusters combined, as pie charts broken down by basic GO organization. To identify significant and highly present GO categories, the most prevalent GO within each cluster was measured for overrepresentation by a standard hypergeometric test. Just below each pie chart title, the most prevalent GO category is listed along with the number of times it appears in the network (labeled as count) and the estimated 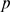-value that is yielded from the hypergeometric test.

The most striking result is seen in cluster 4 where each of the ten genes in the network is categorized with GO:0003677, which represents “DNA binding” under the molecular function ontology. Other significant GO categories in cluster 4 include GO:0005634 (count = 9, 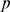 = .0021, “nucleus”, cellular component) and GO:0006334 (count = 8, 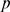 = 3.49e-08, “nucleosome assembly”, biological process). The other interesting result is GO:0016021 in cluster 3 (count = 5, 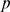 = .0142, “integral to membrane”, cellular component). The names of the genes in clusters 3 and 4 along with their original aliases are provided in Table 9. No significant GO categories were detected in the other clusters. This may be due to a limited number of genes observed in these clusters.

**Table 9:** The names of the genes and their original aliases in clusters 3 and 4.

Cluster 3		Cluster 4	
Gene name	Sanger alias	Gene name	Sanger alias
hhf1	R:A-SNGR-8:5476	hsp16	R:A-SNGR-8:2957
hhf3	R:A-SNGR-8:5202	SPAPB24D3.07C	R:A-SNGR-8:4962
hht1	R:A-SNGR-8:5318	SPBC1347.13C	R:A-SNGR-8:4371
hht2	R:A-SNGR-8:5698	SPBC1348.13	R:A-SNGR-8:2466
hht3	R:A-SNGR-8:5012	SPCC285.05	R:A-SNGR-8:400
hta1	R:A-SNGR-8:916	P07657 (SPMIT.01)	R:A-SNGR-8:4115
hta2	R:A-SNGR-8:4308	P05511 (SPMIT.06)	R:A-SNGR-8:4627
htb1	R:A-SNGR-8:1510	P21535 (SPMIT.07)	R:A-SNGR-8:5907
sap1	R:A-SNGR-8:5023	P21536 (SPMIT.09)	R:A-SNGR-8:3091
SPAC19B12.06c	R:A-SNGR-8:786	P21537 (SPMIT.10)	R:A-SNGR-8:2067

The simulations and real data analyses were performed on a quad-core i7 920 PC overclocked to 4GHz running the Ubuntu Karmic Koala operating system. The timing of the computations varied from several minutes to several hours. AIC and BIC calculation were the most time consuming and multiple models were estimated to determine the best fitting model. The software used to perform these analyses and create the graphs has been made publicly available with further details provided in the following section.

### R Software Package

A new R package, geneARMA [32] that is available on the Comprehensive R Archive Network (CRAN) and licensed under the general public license GPLv3, implements the methods in this paper. This software package provides tools for simulation, estimation, and graphing of the proposed methods in this paper. The estimation and graphics prepared in the real data application of this manuscript were prepared with the geneARMA package.

## Discussion

The proposed mixture model for functional clustering of gene expression profiles provides a flexible framework for estimating the number of mixing components, the periodic means of each component, and the variance-covariance structures. Our approach is useful in comparing the mean expression profiles across different periodic patterns, making it possible to further address the fundamental issues about the interplay between gene expression and biological rhythms. Compared to the existing statistical approaches for temporal gene expression data, our approach has the advantage of fitting a flexible covariance structure into a routine that incorporates mathematical equations for periodic gene expression profiles thereby making the estimation of the mean expression curve more robust to complex covariance phenomena arising in real practice.

We use the Fourier series to model periodicity of the gene expression profiles such as observed in circadian rhythms and cell cycles. The coefficients in the Fourier series provide biologically meaningful interpretations and enables testing of several curve features across different clusters. The gene expression 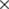 time interaction over a period of time can be tested by evaluating the equality of the slopes 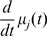 of mean expression profiles among the gene groups.

There is always a balance to be made in the complexity of a model given the amount of data under consideration. Short time course expression profiles typically do not have sufficient data display a periodic signal, and one would typically not use a mixture of sinusoidal signals to estimate the mean curves. Alternative approaches for short time series have been proposed such as [9].

The simulation studies discussed in the Section 3 suggest that the proposed procedure is able to produce sound parameter estimates and increased power compared to the AR(1) model when the true intercorrelation structure of the time-dependent expression data is of a higher order. However, the ARMA covariance structure requires that the gene expression is evaluated at equally spaced times points, which makes it inapplicable when the data are collected irregularly or at gene specific time intervals. Moreover, accurate estimation and classification of gene expression profiles are in need of reasonable approximation of the assumed covariance model to the truth. The simulations also indicate that any parametric methods could be non-robust and produce misleading results when deviation from the true covariance exists. Under these considerations, semi-parametric approaches arise as a promising alternative to the ARMA assumption in the current model [33]. In addition, dimension reduction methods could be integrated into our mixture model to increase the tractability of high dimensional data as the genes are measured over a long time course [34] [35].

Since we usually would not expect periodic expression to exactly follow an ARMA process, the real data analysis was useful to see the effectiveness of the methods in practice. Both the AIC and BIC selected the AR(2) covariance structure suggesting the flexibility in the ARMA parameters provides a improved fit over the more simplistic AR(1) covariance structure. The graphical views of the model fit impressively demonstrate the utility of the proposed method to real datasets.

### Supporting Information

Details of the EM Algorithm are provided as supporting information (Text S1).

## Supporting Information

Text S1 Supporting information EM algorithm.
(0.15 MB PDF)
