# Supplementary material for: Functional Clustering of Periodic Transcriptional Profiles through ARMA(p,q)
Source: PLoS One. 2010 Apr 16;5(4):e9894. doi: 10.1371/journal.pone.0009894 (PMC2855703; doi:10.1371/journal.pone.0009894)
Supplement: Text S1 — Supporting information EM algorithm. (0.15 MB PDF) [file pone.0009894.s001.pdf]

EM Algorithm

In what follows, we give the procedure for estimating parameters in Θ within the EM algorithm framework. In the E-step, the posterior expectation of z_{ij} is evaluated as

$$P_{ij} = E[z_{ij}|\Theta, \boldsymbol{y}_i] = Pr[z_{ij} = 1|\Theta, \boldsymbol{y}_i] = \frac{\omega_j f_{ij}(\boldsymbol{y}_i; \boldsymbol{\mu}_{ij}, \Sigma_i)}{\sum_{j'=1}^{J} \omega_{j'} f_{ij'}(\boldsymbol{y}_i; \boldsymbol{\mu}_{ij'}, \Sigma_i)}. \tag{1}$$

In the M-step, closed form solutions exist for $\boldsymbol{\omega}$ and the parameters in Θ_{μ_j} except for τ and σ^2. Since $\omega_J = 1 - \sum_{j=1}^{J-1} \omega_j$, it is easy to show that for $j = 1, \ldots, J-1$,

$$\frac{\partial \log L_c(\Theta|\boldsymbol{y})}{\partial \omega_j} = \sum_{i=1}^{n} \frac{P_{ij}}{\omega_j} - \sum_{i=1}^{n} \frac{P_{iJ}}{1 - \sum_{j'=1}^{J-1} \omega_{j'}}.$$

By setting the above equation to zero and solving it for ω_j, we have

$$\omega_j = \frac{\sum_{i=1}^{n} P_{ij}(1 - \sum_{j'=1}^{J-1} \omega_{j'})}{\sum_{i=1}^{n} P_{iJ}}. \tag{2}$$

Calculating the sum of both sides of Equation (2) over $j = 1, \ldots, J-1$, we get

$$1 - \omega_J = \frac{\sum_{i=1}^{n} \sum_{j=1}^{J-1} P_{ij}\omega_J}{\sum_{i=1}^{n} P_{iJ}}. \tag{3}$$

Since $\sum_{j=1}^{J-1} P_{ij} = 1 - P_{iJ}$, solving (3) for ω_J, it can be shown that

$$\hat{\omega}_J = \sum_{i=1}^{n} P_{iJ}/n \tag{4}$$

Plugging (4) back into (2), we have $\hat{\omega}_j = \sum_{i=1}^{n} P_{ij}/n$.

Suppose the gene expression trajectory is approximated by the first K orders of the Fourier series, then $\Theta_{\mu_j} = (\boldsymbol{c}_j, \tau_j)$, where $\boldsymbol{c}_j = (\alpha_{0j}, \alpha_{1j}, \beta_{1j}, \ldots, \alpha_{Kj}, \beta_{Kj})$. We have

$$\frac{\partial \log L_c(\Theta|\boldsymbol{y})}{\partial \boldsymbol{c}_j} = \left[\frac{\partial \log L_c(\Theta|\boldsymbol{y})}{\partial \boldsymbol{\mu}_{ij}}\right] \left[\frac{\partial \boldsymbol{\mu}_{ij}}{\partial \boldsymbol{c}_j}\right]. \tag{5}$$

The parameter $\boldsymbol{c}_j$ can be updated by setting (5) to zero. Since

$$\frac{\partial \log L_c(\Theta|\boldsymbol{y})}{\partial \boldsymbol{\mu}_{ij}} = \sum_{i=1}^{n} P_{ij}(\boldsymbol{y}_i - \boldsymbol{\mu}_{ij})^T \Sigma_i^{-1}$$

and $\frac{\partial \mu_{ij}}{\partial \boldsymbol{c}_j} = D_i(\tau_j)$, where

$$D_i(\tau_j) = \begin{pmatrix} 1 & \cos(\frac{2\pi t_{i1}}{\tau_j}) & \sin(\frac{2\pi t_{i1}}{\tau_j}) & \cdots & \cos(\frac{2\pi K t_{i1}}{\tau_j}) & \sin(\frac{2\pi K t_{i1}}{\tau_j}) \\ 1 & \cos(\frac{2\pi t_{i2}}{\tau_j}) & \sin(\frac{2\pi t_{i2}}{\tau_j}) & \cdots & \cos(\frac{2\pi K t_{i2}}{\tau_j}) & \sin(\frac{2\pi K t_{i2}}{\tau_j}) \\ \vdots & \vdots & \vdots & \vdots & \vdots & \vdots \\ 1 & \cos(\frac{2\pi t_{im_i}}{\tau_j}) & \sin(\frac{2\pi t_{im_i}}{\tau_j}) & \cdots & \cos(\frac{2\pi K t_{im_i}}{\tau_j}) & \sin(\frac{2\pi K t_{im_i}}{\tau_j}) \end{pmatrix},$$

we have

$$\hat{\boldsymbol{c}}_j = \left[\sum_{i=1}^{n} P_{ij} D_i(\tau_j)^T \Sigma_i^{-1} D_i(\tau_j)\right]^{-1} \left[\sum_{i=1}^{n} P_{ij} \boldsymbol{y}_i^T \Sigma_i^{-1} D_i(\tau_j)\right].$$

Since the analytical form of the inverse of Σ_i is not available, we use the recursive method proposed by Haddad (2004) to calculate the inverse matrix of ARMA(p, q) through its association with ARMA (p, q-1).

We can write $\Sigma_i = \sigma^2 R_i$, where R_i is the correlation matrix that is entirely determined by the ARMA parameters $\varphi_1, \ldots, \varphi_p, \theta_1, \ldots, \theta_q$. The variance σ^2 can be updated by

$$\hat{\sigma}^2 = \frac{\sum_{i=1}^{n} \sum_{j=1}^{J} P_{ij} (\boldsymbol{y}_i - \mu_{ij})^T R_i^{-1} (\boldsymbol{y}_i - \mu_{ij})}{\sum_{i=1}^{n} m_i}. \tag{6}$$

Again R_i^{-1} can be calculated by the method of Haddad (2004).

Because there are no closed form solutions for τ_j and ARMA parameters $\varphi_1, \ldots, \varphi_p$ and $\theta_1, \ldots, \theta_q$, their estimates are updated using one-step Newton-Raphson method within each iteration. In particular, in the $(\nu + 1)$-th iteration, τ_j can be updated by

$$\tau_j^{\nu+1} = \tau_j^{\nu} - \frac{\frac{\partial}{\partial \tau_j} \log L_c(\Theta|\boldsymbol{y})|_{\Theta=\Theta^\nu}}{\frac{\partial^2}{\partial \tau_j^2} \log L_c(\Theta|\boldsymbol{y})|_{\Theta=\Theta^\nu}}, \tag{7}$$

where

$$\frac{\partial}{\partial \tau_j} \log L_c(\Theta|\boldsymbol{y}) = \sum_{i=1}^{n} P_{ij} (\boldsymbol{y}_i - \mu_{ij})^T \Sigma_i^{-1} \delta_{ij}$$

with δ_{ij} being a $m_i \times 1$ vector whose components

$$\delta_{ijl} = \sum_{k=1}^{K} \left[\alpha_{kj} \sin\left(\frac{2\pi k t_l}{\tau_j}\right) \frac{2\pi k t_l}{\tau_j^2} - \beta_{kj} \cos\left(\frac{2\pi k t_l}{\tau_j}\right) \frac{2\pi k t_l}{\tau_j^2}\right],$$

and

$$\frac{\partial^2}{\partial \tau_j^2} \log L_c(\Theta|\boldsymbol{y}) = \sum_{i=1}^{n} \left[-P_{ij} \delta_{ij}^T \Sigma_i^{-1} \delta_{ij} + P_{ij} (\boldsymbol{y}_i - \boldsymbol{\mu}_{ij})^T \Sigma_i^{-1} \frac{\partial^2}{\partial \tau_j^2} \boldsymbol{\mu}_{ij}\right].$$

Similarly, the parameters $\varphi_1, \ldots, \varphi_p$ and $\theta_1, \ldots, \theta_q$ can be updated by the one-step Newton-Raphson method outlined above. However, there are no analytical forms of the first and the second derivatives of the expected complete data log-likelihood with respect to the φ's and θ's, we use the numerical differentiation method to calculate these quantities (Zeng and Cai, 2005). To ease the presentation of the method, denote the $(p + q)$ dimensional vector $\psi = (\varphi_1, \ldots, \varphi_p, \theta_1, \ldots, \theta_q)$. The first and the second derivatives with respect to the κ-th component in ψ are approximated, respectively, by

$$\frac{E[\log L_c(\Theta_{-\psi}, \psi + h_n e_\kappa|\boldsymbol{y})] - E[\log L_c(\Theta|\boldsymbol{y})]}{h_n}, \tag{8}$$

and

$$\frac{E[\log L_c(\Theta_{-\psi}, \psi + h_n e_\kappa|\mathbf{y})] - 2E[\log L_c(\Theta|\mathbf{y})] + E[\log L_c(\Theta_{-\psi}, \psi - h_n e_\kappa|\boldsymbol{y})]}{h_n^2}, \tag{9}$$

where we use E to represent the posterior expectation of the complete data log-likelihood with respect to w_{ij}, $\Theta_{-\psi}$ denotes the parameters in Θ other than ψ, the $(p+q)$ vector e has unity length with the κ-th component set to 1, and h_n is the bandwith chosen by the investigator. When h_n is small enough, the numerical differentiation approximates the true derivatives adequately. On the other hand, if h_n is too small, the random errors from the numerical computation may deteriorate the results.
